# Deleterious *AHNAK2* Mutation as a Novel Biomarker for Immune Checkpoint Inhibitors in Non-Small Cell Lung Cancer

**DOI:** 10.3389/fonc.2022.798401

**Published:** 2022-03-10

**Authors:** Yanan Cui, Xinyin Liu, Yuemin Wu, Xiao Liang, Jiali Dai, Zhihong Zhang, Renhua Guo

**Affiliations:** ^1^ Department of Oncology, The First Affiliated Hospital of Nanjing Medical University, Nanjing, China; ^2^ Department of Pathology, The First Affiliated Hospital of Nanjing Medical University, Nanjing, China

**Keywords:** NSCLC, *AHNAK2*, immune checkpoint inhibitors, biomarker, immune microenvironment

## Abstract

Immune checkpoint inhibitors (ICIs) have exhibited promising efficacy in non-small cell lung cancer (NSCLC), but the response occurs in only a minority of patients. In clinic, biomarkers such as TMB (tumor mutation burden) and PD-L1 (programmed cell death 1 ligand 1) still have their limitations in predicting the prognosis of ICI treatment. Hence, reliable predictive markers for ICIs are urgently needed. A public immunotherapy dataset with clinical information and mutational data of 75 NSCLC patients was obtained from cBioPortal as the discovery cohort, and another immunotherapy dataset of 249 patients across multiple cancer types was collected as the validation. Integrated bioinformatics analysis was performed to explore the potential mechanism, and immunohistochemistry studies were used to verify it. *AHNAK* nucleoprotein 2 (*AHNAK2*) was reported to have pro-tumor growth effects across multiple cancers, while its role in tumor immunity was unclear. We found that approximately 11% of the NSCLC patients harbored *AHNAK2* mutations, which were associated with promising outcomes to ICI treatments (ORR, p = 0.013). We further found that *AHNAK2* deleterious mutation (del-*AHNAK2* ^mut^) possessed better predictive function in NSCLC than non-deleterious *AHNAK2* mutation (PFS, OS, log-rank p < 0.05), potentially associated with stronger tumor immunogenicity and an activated immune microenvironment. This work identified del-*AHNAK2* ^mut^ as a novel biomarker to predict favorable ICI response in NSCLC.

## Introduction

Lung cancer, histologically differentiated into small cell lung cancer and non–small cell lung cancer, ranks among the most common cause of cancer-related death worldwide. Non-small cell lung cancer (NSCLC) accounts for about 85% of total cases routinely being treated with surgery, chemotherapy, radiation therapy, or targeted therapy. Nowadays, tumor immune checkpoint inhibitors (ICIs), including programmed death receptor 1 (PD-1), PD-ligand 1 (PD-L1), and cytotoxic T-lymphocyte-associated protein 4 (CTLA-4), have revolutionized NSCLC treatment (1, 2). While ICIs have the potential for a durable response, the response occurs in only a minority of patients (3, 4). Therefore, predictive biomarkers of ICI treatment response are required to provide effective therapy. Although tumor mutation burden (TMB), PD-L1 expression, tumor immune microenvironment (TIME), and some specific gene mutations have been reported to be associated with ICI response (5–9), only a few of them have been validated in the clinic and even those validated biomarkers still had their limitations.

*AHNAK2*, a protein consisting of many extremely conserved duplicated fragments, shares extensive homology with *AHNAK1* and can interact with the C-terminal peptides of a variety of channel proteins (10). *AHNAK1* and *AHNAK2* co-localized with vinculin, suggesting that the proteins are components of the costameric network (11). It has previously been observed that *AHNAK2* is overexpressed in many cancers and acts as a poor prognostic biomarker in clear cell renal cell carcinoma (ccRCC), pancreatic ductal carcinoma (PDAC), papillary thyroid carcinoma (PTC), and lung adenocarcinoma (LUAD) (12–14). Several studies showed that *AHNAK2* promotes cell proliferation, migration, invasion, and epithelial–mesenchymal transition in LUAD *via* the MAPK pathway and the TGF-beta/Smad3 pathway (15, 16). A recent study has demonstrated that the expression of *AHNAK2* is related to TIICs (tumor-infiltrating immune cells) (17). However, there is no study illustrating the relationship between *AHNAK2* and immune response.

In this study, we performed an extensive analysis of *AHNAK2* mutation using the previously published immunotherapy cohorts and aimed to explore the predictive value of the *AHNAK2* to ICI response in NSCLC patients.

## Materials and Methods

### Data Download and Processing

**Figure 1:**
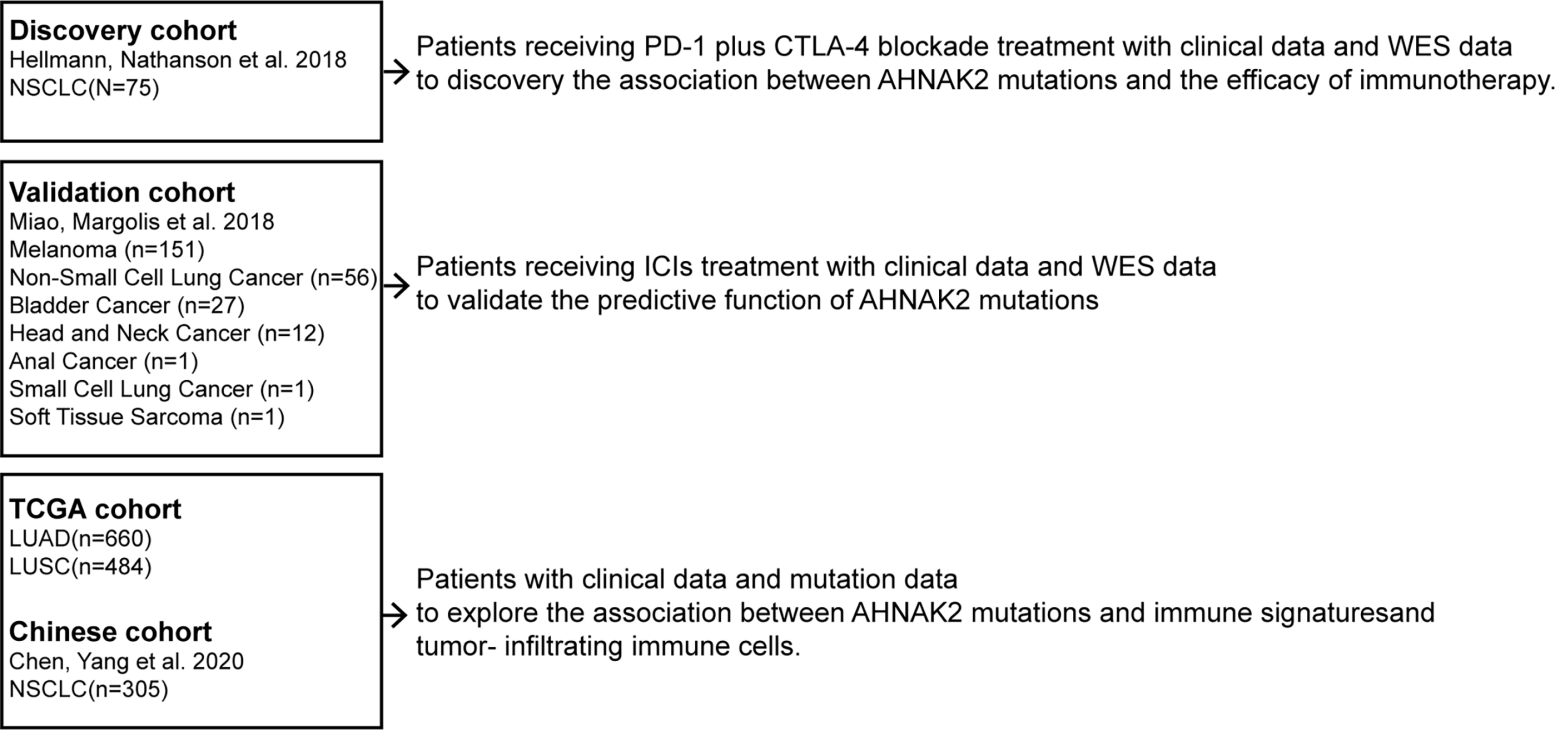
Flow diagram of the study.

The flow diagram of this study is depicted in **Figure 1**. To evaluate the predictive functions of *AHNAK2* in ICI-treated patients, we use the data accessed from the cBioPortal (https://www.cbioportal.org). A total of 75 NSCLC patients who received anti-PD-L1 plus anti-cytotoxic T-cell lymphocyte-4 (anti-CTLA-4) treatment with whole-exome sequencing (WES) data (18) were included in our research as the discovery cohort. The validation cohort consisted of 249 patients across multiple cancer types who had received ICI therapy (19).

To study the mechanism underlying the association between *AHNAK2* mutation and ICI response, we obtained the WES data along with the corresponding mRNA expression data of 513 LUAD and 480 LUSC in TCGA cohort from the UCSC Xena data portal (https://xenabrowser.net). In addition, 305 Chinese NSCLC patients (20) from cBioPortal, referred to as Chinese cohort, were used to validate the findings.

### Clinical Outcomes

The primary clinical outcomes were objective response rate (ORR), progression-free survival (PFS), and overall survival (OS). ORR was assessed with Response Evaluation Criteria in Solid Tumors (RECIST) version 1.1. OS and PFS were measured from the start date of ICI therapy to the date of death or progression, respectively, in the ICI-treated cohort. Patients who had not died or progressed were censored at the date of their last scan. In TCGA cohort, OS and PFS were both assessed from the date of the first diagnosis.

### PROVEAN Protein Database Evaluating the Mutational Impact on Protein Structure

Missense mutations might be deleterious or benign, on account of their effects on protein structure. The PROVEAN tool (http://provean.jcvi.org) was used to predict the function of missense variants (21). The cutoff for PROVEAN scores was set to −2.5 for high balanced accuracy, and we selected those amino acid changes with the PROVEAN score <−2.5, indicating a deleterious effect on protein function.

### Tumor Mutation Characteristics, Tumor Immunogenicity, and TIME Analysis

The cBioPortal online tool was used for visualization of the mutation rates of the top 20 genes and clinical characteristics. TMB was defined as the total number of non-synonymous somatic, coding, base substitution, and indel mutations per megabase (Mb) of the genome examined (22). Neoantigen data in the TCGA cohort were obtained from Thorsson et al. (23).

GSEA was used to associate the gene signatures with *AHNAK2* deleterious mutation (del-*AHNAK2* ^mut^), *AHNAK2* non-deleterious mutation (non-del-*AHNAK2* ^mut^), and *AHNAK2* wild type (*AHNAK2* ^wt^). The normalized enrichment score (NES) is the primary statistic for examining gene set enrichment results. The single-sample gene set enrichment analysis (ssGSEA) method was applied to quantify the enrichment levels of the immune signatures in each sample using the “GSVA” R package (24). Twenty-nine characteristic immune signatures were obtained from a previous study (25).

The levels of TILs were calculated by analyzing the data from Saltz et al., who used deep learning-based lymphocyte classification to estimate the H&E-stained images from TCGA dataset (26). The infiltration statuses of twenty-two immune cells were analyzed using the CIBERSORT web portal (https://cibersort.stanford.edu/) (27).

### Human Sample Collection and Immunohistochemistry

We collected five cases with two pulmonary nodules from the First Affiliated Hospital of Nanjing Medical University, both of which were confirmed as primary NSCLC by pathology. All samples were sequenced through whole-exome sequencing. Interestingly, in each case, one primary lung cancer was confirmed with *AHNAK2* mutation and the other was confirmed without *AHNAK2* mutation. Anti-CD3, anti-IFNG, and anti-GZMB immunohistochemistry (IHC) staining assays were performed on the tissue sections. The following antibodies were used: anti-CD3 (Servicebio, Wuhan, China, GB111337), anti-GZMB (Servicebio, GB14092), anti-IFNG (Abcam, Cambridge, CA, USA, ab231036). The digital quantitation of immunohistochemistry staining was conducted with HALO image analysis software (Indica Labs, Albuquerque, NM, USA).

### Statistical Analyses

All analyses and statistical tests were carried out with the R software (version 3.6.1). Univariate Kaplan–Meier survival analysis was performed, and the survival curves were compared using the log-rank test. Fisher’s exact test was used to assess the enrichment of *AHNAK2* status with response (ORR). All comparisons were conducted using the Wilcoxon test. Statistical significance was set as 2-sided p  <  0.05 for all comparisons.

## Results

### Association Between *AHNAK2* Mutation and Better Clinical Benefit to ICIs in the Discovery Cohort

**Figure 2:**
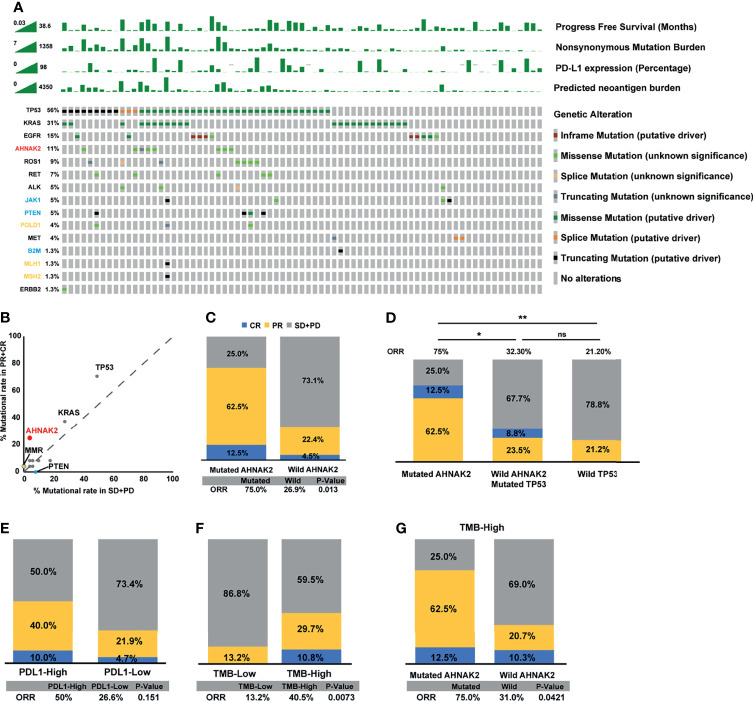
*AHNAK2* mutations are significantly associated with better clinical benefit to ICIs in the discovery cohort. **(A)** Stacked plots show mutational burden, PD-L1 expression, and predicted neoantigen burden (histogram, top); mutations in TP53, KRAS, EGFR, ROS1, RET, ALK, MET, ERBB2 (genes recommended to tested according NCCN guidelines), and JAK1, PTEN, POLD1, B2M, MLH1, MSH2 (genes had been reported to affect the efficacy of immunotherapies; yellow represents positive predictors of immunotherapy, while blue represents negative predictors of immunotherapy) (tile plot, middle) and mutational marks (right). **(B)** Scatter diagram displays the mutational rate in patients having achieved objective response or progressive disease. MMR: mismatch repair genes (MLH1, MSH2). **(C)** Histogram depicting proportions of ORR in mutated *AHNAK2* and wild *AHNAK2* patients. **(D)** Histogram depicting proportions of ORR in mutated *AHNAK2*, mutated *TP53*/wild *AHNAK2*, and wild *TP53* patients. **(E)** Histogram depicting proportions of ORR in PDL1-High and PDL1-Low patients. **(F)** Histogram depicting proportions of ORR in mutated TMB-High and TMB-Low patients. **(G)** Histogram depicting proportions of ORR in mutated *AHNAK2* and wild *AHNAK2* among TMB-High patients (Mann–Whitney U test; ns, not significant; *p < 0.05, **p < 0.01).

We collected 75 NSCLC patients who received ICI treatment with WES data as the discovery cohort. The genomic mutational landscape from the discovery cohort is displayed in **Figure 2A** (including genes as suggested by NCCN guidelines and other oncogenes which had been reported to affect the efficacy of immunotherapies (28–31)). In agreement with previous studies, higher mutational rates of *TP53*, *KRAS*, and MMR-related genes were shown in responders relative to non-responders. On top of that, *AHNAK2* mutation was also observed to be enriched in responders (**Figure 2B**). The frequency of *AHNAK2* mutation was up to 11%, indicating that it was not a rare mutation in NSCLC. We found that the mutation of *AHNAK2* was statistical significantly associated with higher ORR (**Figure 2C**), suggesting that *AHNAK2* mutation might predict an improved clinical outcome for NSCLC patients treated with ICIs.

Interestingly, we found that all patients with *AHNAK2* mutation harbored *TP53* mutation in the discovery cohort. As *TP53* mutations are known to be correlated with response to immunotherapy, we compared ICI response between patients with *TP53* mutation only and patients with both *TP53* and *AHNAK2* mutation. As shown in **Figure 2D**, patients with *AHNAK2* and *TP53* co-mutations achieved a higher ORR than patients with *TP53* mutation only. Besides, among patients without *AHNAK2* mutation, *TP53* was not a statistically significant predictor. These results implied that clinical benefits of ICIs were even more remarkable in patients with *AHNAK2* mutation than in those with *TP53* mutation.

In addition, we compared the predictive value of *AHNAK2* mutation with other widely recognized ICI biomarkers. Here, we found that patients with high PD-L1 expression (≥50% percentage) had higher ORR than those with low PD-L1 expression (<50% percentage), while this difference was not statistically significant (**Figure 2E**). Patients with high TMB level were more likely to benefit from the immunotherapy, which was also observed here (**Figure 2F**). Notably, *AHNAK2* mutation could identify patients with better response to immunotherapy among TMB-high patients (**Figure 2G**), indicating that the combination of *AHNAK2* mutation and TMB level could better stratify patients to identify those who are likely to benefit most from immunotherapy.

### Del-*AHNAK2* ^mut^ Is a Predictive, Not Prognostic, Biomarker of ICI Benefit

**Figure 3:**
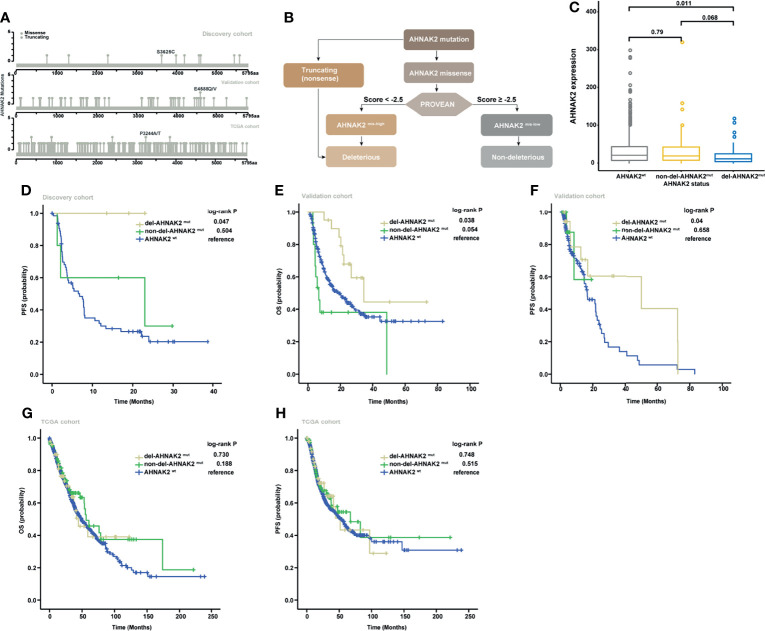
Validation of the predictive significance of *AHNAK2* in pan-cancer. **(A)** Lollipop plots showing the locus distribution of mutations across the *AHNAK2*-altered patient cohorts. **(B)** Flow diagram of the identification of del-*AHNAK2*mut and non-del-*AHNAK2*mut by mutational forms and PROVEAN. **(C)** Kaplan–Meier curves of PFS in the discovery cohort classified by *AHNAK2* mutations. **(D)** Kaplan–Meier curves of OS in the validation cohort classified by *AHNAK2* mutations. **(E)** Kaplan–Meier curves of PFSS in the validation cohort classified by *AHNAK2* mutations. **(F)** Kaplan–Meier curves of OS in the TCGA cohort classified by *AHNAK2* mutations. **(G)** Kaplan–Meier curves of PFS in the TCGA cohort classified by *AHNAK2* mutations.

As illustrated in **Figure 3A**, most of the *AHNAK2* alterations were missense mutations, which can be partitioned into deleterious or benign, depending on whether they affect protein structures or not. To exclude the benign mutations that generally do not affect the protein function, the PROVEAN tool was applied to distinguish the missense mutations of *AHNAK2* with a high probability of impact on protein structures with a low probability (abbreviated as *AHNAK2* ^mis-high^ and *AHNAK2* ^mis-low^, respectively). Since both truncating and mis-high mutations affect the biological function of the involved genes, we therefore grouped these two kinds of alterations as del-*AHNAK2* ^mut^. Relatively, patients with mis-low mutations of *AHNAK2* were defined as non-del-*AHNAK2* ^mut^ whereas the patients without any *AHNAK2* mutations were categorized as the control group (*AHNAK2* ^wt^). A schematic of the workflow is shown in **Figure 3B**. As shown in **Figure 3C**, the patients harboring deleterious *AHNAK2* mutations, not non-deleterious *AHNAK2* mutations, possessed a trend of better PFS than the patients with *AHNAK2* ^wt^ in the discovery cohort.

To further assess the predictive value of deleterious *AHNAK2* mutations, we explored another pan-cancer immunotherapy cohort (19) as an independent external validation. In this validation cohort, the overall mutation frequency of *AHNAK2* was 14% with melanoma (17.2%) ranking the most prevalent cancer type followed by non-small cell lung cancer (12.5%) and bladder cancer (7.4%). Consistent with the results described above, the patients harboring deleterious *AHNAK2* mutation achieved better survival from ICI treatment compared with those without *AHNAK2* mutation (**Figures 3D, E** **)**. Taking TCGA cohort as a comparison, which includes the NSCLC patients without receiving immunotherapy, we found no significant survival differences between del-*AHNAK2* ^mut^ and *AHNAK2* ^wt^ (**Figures 3F, G** **)**. These results suggested that del-*AHNAK2* ^mut^ was not linked with prognosis but was associated with significant immunotherapeutic efficacy independently, which delineated the potential predictive value of del-*AHNAK2* ^mut^ in immunotherapy against NSCLC.

### Association Between *AHNAK2* ^mut^ and Higher Tumor Immunogenicity

**Figure 4:**
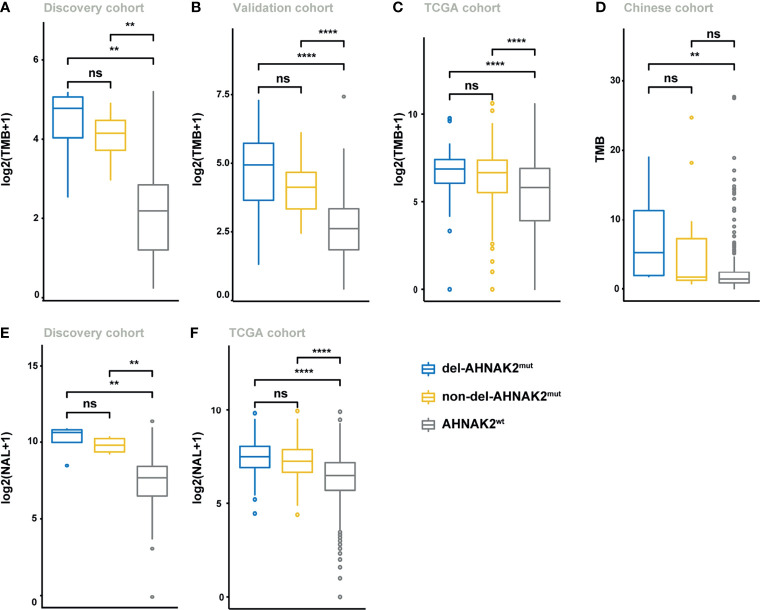
Del-*AHNAK2* ^mut^ was associated with the enhanced tumor immunogenicity. **(A–D)** Comparison of the levels of TMB in patients from discovery cohort **(A)**, validation cohort **(B)**, TCGA cohort **(C)**, and Chinese cohort **(D)** classified by *AHNAK2* mutations (del-*AHNAK2* ^mut^, non-del-*AHNAK2* ^mut^, and no *AHNAK2* mutation). **(E, F)** Comparison of the levels of NAL in patients from discovery cohort **(E)** and TCGA cohort **(F)** classified by *AHNAK2* mutations (del-*AHNAK2* ^mut^, non-del-*AHNAK2* ^mut^, and no *AHNAK2* mutation). (Mann–Whitney U test; ns, not significant; **p < 0.01, ****p < 0.0001).

To investigate the potential mechanism by which *AHNAK2* mutations influence the sensitivity of ICI therapy, we first analyzed the mutational burden (TMB) and neoantigen load (NAL). As shown in **Figure 4A**, higher TMB levels were observed in *AHNAK2* ^mut^ tumors in the discovery cohort, which was verified in the validation cohort, TCGA cohort, and Chinese cohort (**Figures 4B–D**). In addition, the level of NAL was significantly higher in the *AHNAK2* ^mut^ tumors as well, relative to the *AHNAK2* ^wt^ group (**Figures 4E, F** **)**. These results suggested that *AHNAK2* ^mut^ tumors were strongly related to enhanced tumor immunogenicity, firmly supporting its predictive value for ICI therapy.

### Impact of del-AHANK2^mut^ on Activated Immune Microenvironment

**Figure 5:**
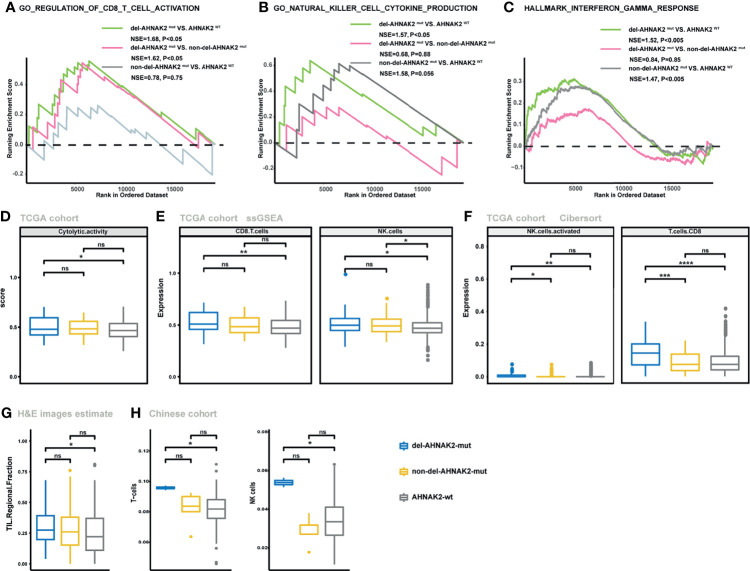
The mutation status of *AHNAK2* was associated with the activated immune microenvironment. **(A–C)** GSEA of signatures related to CD8 T cell activation **(A)**, NK cell activation **(B)**, and IFN-γ **(C)** in comparisons between NSCLC samples with del-*AHNAK2* ^mut^, non-del-*AHNAK2* ^mut^, and no *AHNAK2* mutation. **(D)** Comparison of the cytolytic activity score between *AHNAK2* ^wt^, del-*AHNAK2* ^mut^, and non-del-*AHNAK2* ^mut^ tumors by the ssGSEA method based on TCGA RNA-sequencing data. **(E)** Comparison of the infiltration degree of immune cells by the ssGSEA method based on RNA-sequencing data between *AHNAK2* ^wt^, del-*AHNAK2* ^mut^, and non-del-*AHNAK2* ^mut^ tumors. **(F)** Comparison of the infiltration degree of immune cells by the CIBERSORT analysis. **(G)** Comparison of the TIL regional fractions based on estimates from processing diagnostic H&E images between *AHNAK2* ^wt^, del-*AHNAK2* ^mut^, and non-del-*AHNAK2* ^mut^ tumors. **(H)** Comparison of the infiltration degree of immune cells in the Chinese NSCLC cohort. (Mann–Whitney U test; ns, not significant; *p < 0.05, **p < 0.01, ***p < 0.001, ****p < 0.0001)

Despite the undifferentiated levels of TMB and NAL observed in the del-*AHNAK2* ^mut^ and non-del-*AHNAK2* ^mut^ groups, the potential mechanisms to immune activation might be dissimilar between these two groups. Cytotoxic T lymphocytes (CD8+ T cells and NK cells) were major mediators of the innate and adaptive antitumor immune response and can use cytotoxic granules containing perforin and granzymes to lyse cancer cells. GSEA revealed prominent enrichment of signatures related to CD8+ T cell and NK cell activation in the del-*AHNAK2* ^mut^ group, compared with the non-del-*AHNAK2* ^mut^ and *AHNAK2* ^wt^ groups (**Figures 5A, B** **)**. In addition, enhanced IFN-γ signaling was also observed in the del-*AHNAK2* ^mut^ group (**Figure 5C**). Previous studies have implicated that the efficiency of anti-PD-L1 and anti-CTLA-4 therapy was dependent on IFN-γ. Produced by cytotoxic immune cells, IFN-γ could facilitate CD8+ T cell and NK cell filtration (32). Next, to test whether del-*AHNAK2* ^mut^ was associated with cytotoxic T lymphocyte function and infiltration, the ssGSEA method was utilized to evaluate the immune status of each patient by analyzing the expression profiles of 29 immune signatures (25). As expected, we found that del-*AHNAK2* ^mut^ tumors expressed higher cytolytic activity scores (**Figure 5D**), indicating that del-*AHNAK2* ^mut^ might liberate CTL cell effector functions. Beyond this, the del-*AHNAK2* ^mut^ tumors were also characterized by more CD8+ T cell and NK cell infiltrations according to the ssGSEA results (**Figure 5E**). To cross-examine the above results with different methods of evaluating immune cells, we analyzed the distribution of 22 immune cells based on the CIBERSORT method, observing an increase of CD8+ T cells and NK cells in the tumors with del-*AHNAK2* ^mut^, relative to those with non-del-*AHNAK2* ^mut^ and *AHNAK2* ^wt^ (**Figure 5F**). According to the TIL fraction data estimated from hematoxylin and eosin-stained (H&E-stained) slides (26), we found that the TIL fraction of the del-*AHNAK2* ^mut^ tumors was higher than that of the *AHNAK2* ^wt^ tumors (**Figure 5G**). To further validate the above results, we also analyzed the RNA-sequencing data from the Chinese cohort and obtained similar findings for the high T cell and NK cell infiltration in del-*AHNAK2* ^mut^ tumor (**Figure 5H**).

### Immunohistochemistry Confirmed the Association Between del-AHANK2^mut^ and Activated Immune Microenvironment

**Figure 6:**
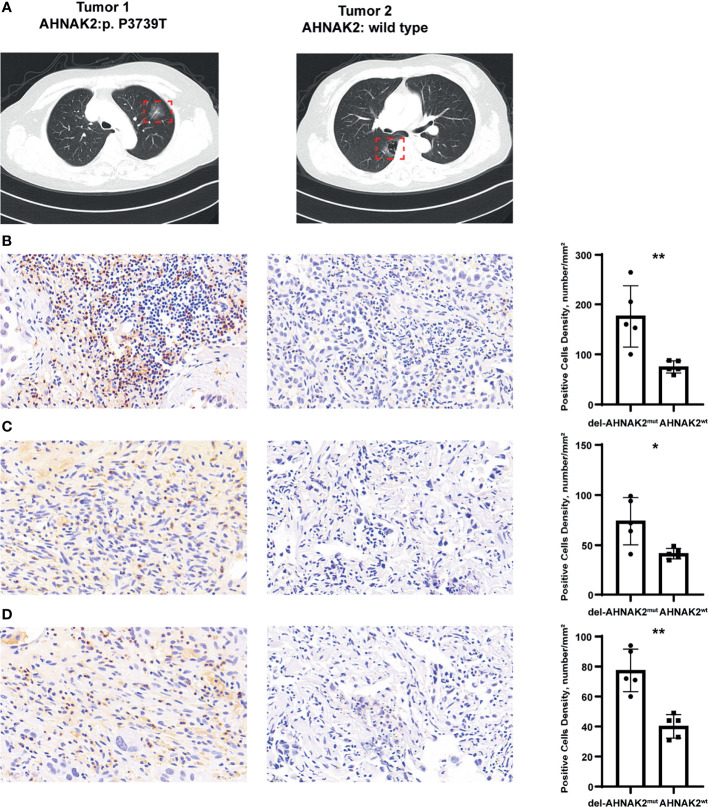
Immunohistochemical staining to evaluate the association between del-*AHNAK2* ^mut^ and activated immune microenvironment. **(A)** Computed tomography (CT) imaging of the representative MPLC patient. **(B–D)** Quantitative immunohistochemistry (IHC) analysis of CD3 **(B)**, GZMB **(C)**, and IFNG **(D)** protein expression in the del-*AHNAK2* ^mut^ and *AHNAK2* ^wt^ groups (n = 5/group). Representative micrographs of these samples are shown. Data represent the mean ± SD. (Mann–Whitney U test; *p < 0.05, **p < 0.01).

We collected 5 patients with multiple lung cancer (MPLC) from the First Affiliated Hospital of Nanjing Medical University, and each patient owned two primary tumors (**Figure 6A**). After surgical treatment, a total of 10 resected tumors from the 5 patients were processed for whole-exome sequencing. Many studies have revealed that in the context of identical constitutional genetic background and environmental exposure, different lung cancers in the same individual have distinct genomic profiles and can be driven by distinct molecular events (33, 34). In our study, few shared mutations were detected between two tumors from the same patient by performing whole-exome sequencing (**Figures S1A, B**). Interestingly, in each patient from our study, one tumor was conformed with deleterious *AHNAK2* mutation and the other was conformed without *AHNAK2* mutation (**Figure S1A**). In this unique model, tumors with different mutation statuses of *AHNAK2* came from the same patient, so some interference caused by genetic background and lifestyle among patients can be reduced to a large extent, enabling us to better explore the association between del-*AHNAK2* ^mut^ and the immune environment. As shown in **Figure 6B**, immunohistochemical (IHC) staining of CD3 confirmed the presence of tumor-infiltrating T cells in the del-*AHNAK2* ^mut^ tumors. In addition, CD3, IFNG, and GZMB were markedly increased in del-*AHNAK2* ^mut^ tumors compared to *AHNAK2* ^wt^ tumors (**Figures 6C, D** **)**.

To sum up, del-*AHNAK2* ^mut^ positively correlated with activated CTL effector functions, IFN-γ signaling, and infiltration of TIILs, which contributed to the activated immune microenvironment.

## Discussion

In recent years, immunotherapy has become a critical milestone of oncology. The growing types of immunotherapy treatments are put into clinical application. Meanwhile, the selection of patients who could get benefit from immunotherapy has also become a formidable clinical problem. Given that the PD-L1 expression or TMB level could not be sufficient to select patients who may benefit the most from immunotherapy, many studies dedicated to identify more precise biomarkers to predict the efficacy of immunotherapy and genetic alterations are among the best-studied (35). For example, gene mutation biomarkers such as *POLE*, *ARID1*, and *FGFR4* increase the TMB level, expression of PD-L1, and activation of the cellular immunity CD8+ tumor infiltration level (36–38). However, these results were not imperfect.

*AHNAK1* and *AHNAK2* are two members of the *AHNAK* family (10), and several studies have strived to decode their function. *AHNAK* can serve as a structural scaffold to mediate the assembly of multiprotein complexes. Meanwhile, significant evidence has been provided to characterize the role of *AHNAK* in membrane repair and calcium channel regulation (39, 40). In recent studies, *AHNAK1* has also been proved as a tumor suppressor in several cancers, including colorectal cancer, gastric cancer, breast cancer, glioma, and lung cancer (41–45). However, as a homologous gene, *AHNAK2* appears to play the opposite function promoting cell proliferation, migration, invasion, and epithelial–mesenchymal translation. *AHNAK2* expression negatively correlated with activated CD8+ T cell infiltration. We thus posit that the dysfunction of *AHNAK2* may contribute to tumor immunity and improved outcomes in ICI-treated NSCLC.

To the best of our knowledge, this is the first report to propose the predictive value of the *AHNAK2* in ICI treatment. In our study, we demonstrated that patients harboring *AHNAK2* mutations were likely to have a better response to ICI treatment. Importantly, among all kinds of *AHNAK2* alterations, compared to non-deleterious, deleterious mutation might exhibit more predictive power. Del-*AHNAK2* ^mut^ was closely correlated with the higher TMB and NAL level and activation of the NK cells, T cells, and IFN-γ signaling, which in turn initiate a therapeutic immune response in NSCLC.

A major limitation of the study is the limited sample size. *AHNAK2* mutation was not included in the routine gene panel; it could only be detected with whole exon sequencing. So far, only two public immunotherapy studies have used the whole exon sequencing, which has been included in our study as the discovery cohort and validation cohort. To compensate for this limitation, RNA-seq data from the TCGA cohort and Chinese cohort were used to verify the association between del-*AHNAK2* ^mut^ and activated immune microenvironment. Further studies are needed to decrypt the effect of *AHNAK2* in cancer immunotherapy. In addition, while our results suggest that del-*AHNAK2* ^mut^ may play a role in the regulation of the tumor microenvironment, no specific molecular mechanisms were determined. An earlier report raised a potential mechanism by which *AHNAK1* affects CTL function by disrupting calcium entry formation (46). In our study, we also found that *AHNAK2* loss-of-function mutation could liberate CTL (CD8+ T cells and NK cells) effector function. As the homolog gene of *AHNAK1*, whether *AHNAK2* can also influence the calcium entry which in turn regulates TIILs remains a subject for future research.

In conclusion, we proposed that del-*AHNAK2* ^mut^ could serve as a novel biomarker for NSCLC patients with ICI treatment. Predictive biomarkers to ICIs, such as the TMB level, NAL level, and TIICs, are closely associated with deleterious *AHNAK2* mutations.

## Data Availability Statement

The original contributions presented in the study are included in the article/**Supplementary Material**. Further inquiries can be directed to the corresponding authors.

## Ethics Statement

Written informed consent was obtained from the individual(s) for the publication of any potentially identifiable images or data included in this article.

## Author Contributions

YC: conceptualization, methodology, software, data curation, writing—original draft preparation. XyL: writing—reviewing and editing. YW: data curation. XL: methodology. JD: data curation. ZZ and RG: supervision, project administration. All authors contributed to the article and approved the submitted version.

## Funding

This work was supported by grants from the National Natural Science Foundation of China (81972188) and the Medical Important Talents of Jiangsu Province (ZDRCA2016024).

## Conflict of Interest

The authors declare that the research was conducted in the absence of any commercial or financial relationships that could be construed as a potential conflict of interest.

## Publisher’s Note

All claims expressed in this article are solely those of the authors and do not necessarily represent those of their affiliated organizations, or those of the publisher, the editors and the reviewers. Any product that may be evaluated in this article, or claim that may be made by its manufacturer, is not guaranteed or endorsed by the publisher.
