# Supplementary material for: Deleterious AHNAK2 Mutation as a Novel Biomarker for Immune Checkpoint Inhibitors in Non-Small Cell Lung Cancer
Source: Front Oncol. 2022 Mar 10;12:798401. doi: 10.3389/fonc.2022.798401 (PMC8960743; doi:10.3389/fonc.2022.798401)

Supplementary Materials

**Deleterious *AHNAK2* mutation as a novel biomarker for immune checkpoint inhibitors in non-small cell lung cancer**

Yanan Cui 1†, Xinyin Liu 1†, Yuemin Wu1†, Xiao Liang 1, Jiali Dai 1, Zhihong Zhang2*, Renhua Guo 1*

Affiliations:

1 Department of Oncology, The First Affiliated Hospital of Nanjing Medical University, Nanjing, China.

2 Department of Pathology, The First Affiliated Hospital of Nanjing Medical University, Nanjing, China.

†These authors have contributed equally to this work.

Corresponding Author: Renhua Guo, Department of Medical Oncology, the First Affiliated Hospital of Nanjing Medical University, 300 Guangzhou Road, Nanjing, Jiangsu Province, China; Email: rhguo@njmu.edu.cn; Zhihong Zhang, Department of Pathology, The First Affiliated Hospital of Nanjing Medical University, 300 Guangzhou Road, Nanjing, China; Tel: +862568217512, Email: zhangzh@njmu.edu.cn.

Legend

Table S1. Baseline characteristics of the 5 multiple lung cancer patients.

Figure S1. Mutational profiles of the 5 multiple lung cancer patients.

A. Top 50 mutation rate genes.

B. Shared mutation counts in each patient.

Table S1.

| **Patient ID** | **Gender** | **Age** | **Ethnicity** | **Smoking history** | **Tumor ID** | **Size(cm)** | **Location** | **Histology** |
| --- | --- | --- | --- | --- | --- | --- | --- | --- |
| 01 | Female | 69 | Han Chinese | Non-smoker | 01-01 | 1.3 | RLL | IAC |
|  |  |  |  |  | 01-02 | 0.8 | RUL | IAC |
| 02 | Female | 50 | Han Chinese | Non-smoker | 02-01 | 0.9 | RUL | MIA |
|  |  |  |  |  | 02-02 | 0.6 | RUL | MIA |
| 03 | Male | 61 | Han Chinese | Non-smoker | 03-01 | 1.5 | LUL | IAC |
|  |  |  |  |  | 03-02 | 0.6 | RUL | MIA |
| 04 | Female | 60 | Han Chinese | Non-smoker | 04-01 | 2.8 | LUL | IAC |
|  |  |  |  |  | 04-02 | 1.3 | RLL | IAC |
| 05 | Male | 59 | Han Chinese | Non-smoker | 05-01 | 1.5 | LLL | IAC |
|  |  |  |  |  | 05-02 | 2 | LUL | IAC |

LLL left lower lobe, LUL left upper lobe, RLL right lower lobe, RUL right upper lobe

Figure S1.


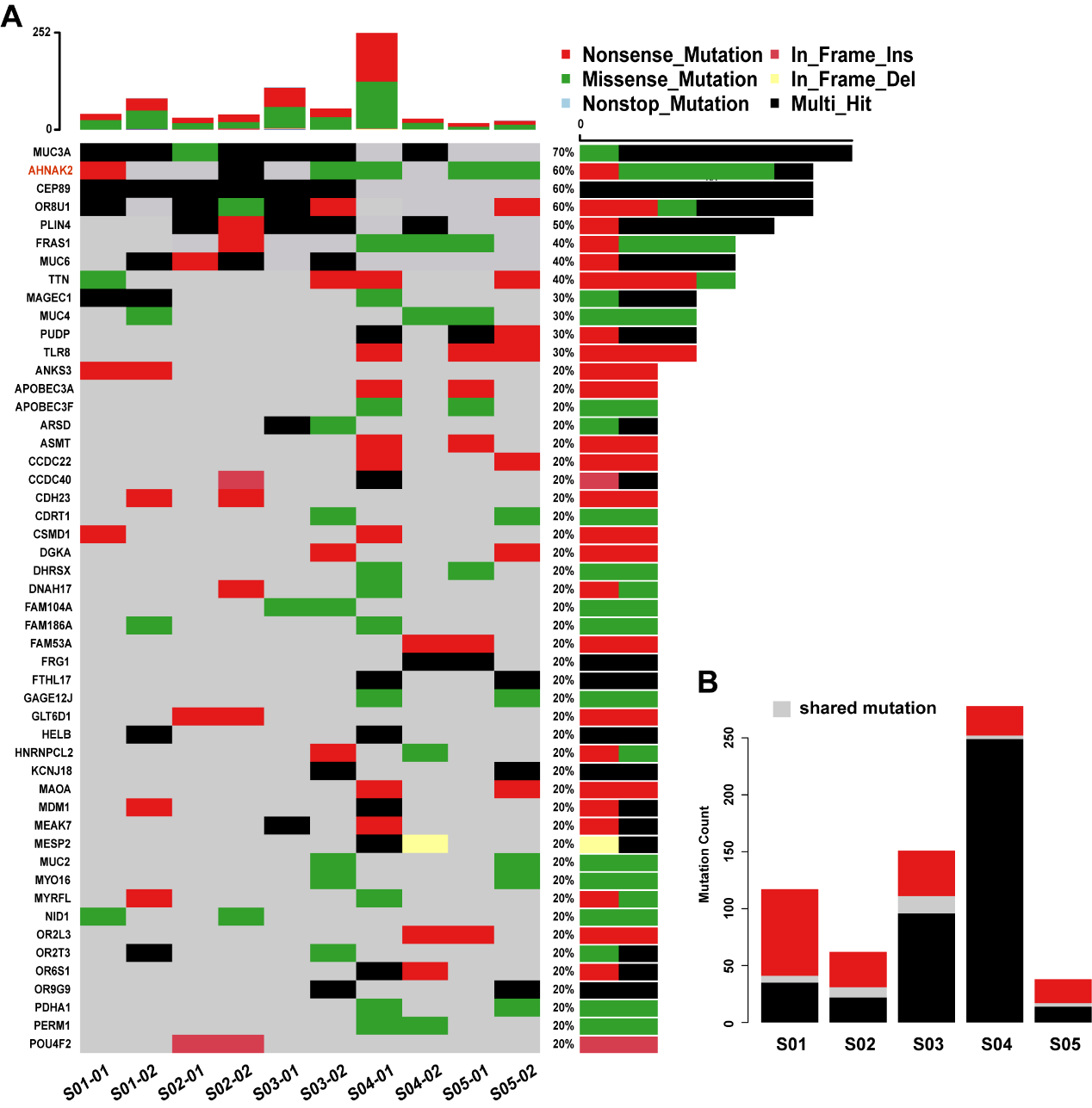

Supplement: Supplementary file 1 [file DataSheet_1.docx]
